# Multi-Dimensional Filler Design for Enhanced Thermal Conductivity and Tunable Dielectric Properties in Natural Rubber Composites

**DOI:** 10.3390/polym18091074

**Published:** 2026-04-29

**Authors:** Yu Li, Qihan Cui, Yining Wang, Yuanqin Gao, Xianhua Hu, Xueqing Liu, Yumin Xia, Lan Cao, Yuwei Chen

**Affiliations:** 1 Key Laboratory of Advanced Rubber Material, Ministry of Education, Qingdao University of Science and Technology, Qingdao 266042, Chinalancao@qust.edu.cn (L.C.); 2 Key Laboratory of Optoelectronic Chemical Materials and Devices, Ministry of Education and Flexible Display Materials and Technology Co-Innovation Centre of Hubei Province, Jianghan University, Wuhan 430056, China; xqliu@jhun.edu.cn; 3 State Key Laboratory for Modification of Chemical Fibers and Polymer Materials, College of Materials Science and Engineering, Donghua University, Shanghai 201600, China; xym@dhu.edu.cn

**Keywords:** thermal conductivity, dielectric properties, filler synergy, composites

## Abstract

Modern electronics demand materials that simultaneously manage heat and provide electromagnetic responses due to high integration and multifunctionality. Therefore, polymer composites with high thermal conductivity and tunable dielectric properties are critical for next-generation electronic devices. Here, natural rubber (NR) was engineered with multi-dimensional fillers—hexagonal boron nitride (h-BN), halloysite nanotubes (HNTs), poly(3-hydroxybutyrate-co-4-hydroxyvalerate) (P34HB), and multi-walled carbon nanotubes (MWCNTs)—to systematically tailor thermal, dielectric, and mechanical properties. Synergistic combinations of h-BN and MWCNTs form an effective three-dimensional thermal network, while HNTs and MWCNTs generate highly effective phonon pathways, achieving a peak thermal conductivity of 0.287 W/(m·K). Dielectric tunability is enabled via percolating h-BN/MWCNT networks, where interfacial polarization allows broad-frequency modulation of the dielectric constant. MWCNTs also regulate curing behavior and provide mechanical reinforcement. In contrast, phase separation between P34HB and NR disrupts the filler network, enabling good electrical insulation while retaining partial thermal pathways, whereas weak interfacial bonding in HNT/MWCNT composites constrains mechanical enhancement. This study demonstrates a systematic multi-dimensional filler strategy enabling tunable thermal and dielectric properties in NR composites and provides a versatile platform for multifunctional polymer materials in flexible and wearable devices.

## 1. Introduction

With the continuous advancement of modern electronic devices toward high integration and miniaturization, increasing power densities have made heat dissipation a critical challenge for long-term operational stability and reliability [1,2]. Developing materials with high thermal conductivity is therefore of paramount importance [3,4]. Polymer matrix composites have attracted significant attention due to their light weight, easy processing, low cost, and excellent electrical insulation, finding widespread applications in electronic packaging [5], thermal interface materials [6], and flexible electronics [7].

Thermally conductive polymer composites are broadly classified into intrinsically conductive polymers [8,9] and filler-based composites [10,11]. Intrinsically conductive polymers enhance heat transfer by achieving highly ordered molecular chain arrangements through molecular design [12], liquid crystal formation [13], or external field orientation [14], thereby reducing phonon scattering, but these approaches often involve complex synthesis and processing. Filler-based thermally conductive polymers incorporate metals [15], ceramics [16], or carbon materials [17], exploiting the intrinsic high thermal conductivity of fillers to enhance heat transport, offering simpler fabrication and lower cost. However, single-filler systems often struggle to simultaneously optimize thermal and dielectric properties, and simply increasing filler content leads to agglomeration, hindering effective phonon transport [18].

To address these issues, researchers have begun exploring hybrid strategies combining fillers of different dimensions. By leveraging the geometric complementarity of zero-, one-, and two-dimensional fillers, more efficient three-dimensional thermal networks can be constructed within the matrix. For instance, Yu et al. incorporated GNPs and MWCNTs into polycarbonate, achieving synergistic enhancement in thermal conductivity [19]. Zeng et al. fabricated a three-dimensional anisotropic BN/MWCNT/epoxy composite exhibiting synergistic optimization of thermal conductivity and electromagnetic shielding at low loadings [20]. Alignment of fillers using external fields [21,22], sacrificial template methods [23], and grafting of polymer brushes onto filler surfaces [24] have also been successfully employed.

Beyond experimental approaches, theoretical and computational modeling has become essential for understanding transport mechanisms in carbon-based polymer composites. For instance, multiscale frameworks that bridge atomistic simulations with continuum mechanics enable accurate prediction of effective thermal conductivity as a function of filler orientation, aspect ratio, and interfacial thermal resistance [25,26]. Meanwhile, machine learning methods have also been employed to accelerate material design [25,26]. For electrically conductive composites, percolation theory provides a fundamental framework to describe the insulator-to-conductor transition [27]. Additionally, Maxwell–Wagner–Sillars (MWS) interfacial polarization is widely used to interpret frequency-dependent dielectric responses [28].

Despite significant progress achieved by existing strategies for polymer-based thermally conductive composites, the introduction of carbon-based conductive fillers often compromises electrical insulation by forming electron percolation networks, limiting their application in electronic packaging [29]. To address this issue, two main strategies have emerged: employing intrinsically insulating thermally conductive fillers such as BN and Al_2_O_3_ to construct thermal networks via filler hybridization or external field orientation while maintaining insulation [30], or applying surface coatings on conductive fillers to block electron pathways while preserving phonon transport [31]. In this work, we explore two additional approaches: first, HNT-induced alignment of MWCNTs in the NR matrix via hydrogen bonding to create oriented one-dimensional phonon pathways; second, P34HB phase segregation to decouple electrical and thermal conductivities, achieving a unique “conductivity-off, thermal-on” state. This study systematically investigates the synergistic effects of multi-dimensional fillers in natural rubber (NR) composites. Two-dimensional hexagonal boron nitride (h-BN), one-dimensional multi-walled carbon nanotubes (MWCNTs) and halloysite nanotubes (HNTs), along with biobased P34HB, are incorporated into the NR matrix via internal mixing to construct hierarchical thermal and dielectric networks. By studying single fillers and various hybrid systems, this work aims to elucidate the synergistic mechanisms among fillers of different dimensions and provide a theoretical basis for designing high-performance NR-based thermally conductive composites.

Nevertheless, the thermal conductivity of the resulting composites (maximum of 0.287 W/(m·K)) remains considerably lower than that of conventional thermal interface materials (>1–2 W/(m·K)), which are typically required for advanced electronic devices. Therefore, this study should be considered as a proof-of-concept demonstration of multi-dimensional filler synergy in natural rubber, rather than a practical solution for high-performance thermal management. Future work will focus on further enhancing thermal conductivity through network optimization and filler functionalization.

## 2. Materials and Methods

### 2.1. Materials

Natural rubber (NR) was purchased from Tianjin Changli Rubber Co., Ltd. (Tianjin, China). Hexagonal boron nitride (h-BN), with a diameter of 300 nm, was purchased from Shanghai Yunfu Nanotechnology Co., Ltd. (Shanghai, China). Multi-walled carbon nanotubes (MWCNTs), with a tube diameter of 3–8 nm, were purchased from Foshan Geruifang New Energy Co., Ltd. (Foshan, China). Poly(3-hydroxybutyrate-co-4-hydroxyvalerate) (P34HB) (M.W. 800 kDa) was purchased from Maiderfa Biomaterials Co., Ltd. (Shenzhen, China). Halloysite nanotubes (HNTs), with a tube diameter of 40–70 nm, were purchased from Guangdong Jina New Material Technology Co., Ltd. (Jieyang, China).

### 2.2. Filler Preparation

All fillers were dried at 60 °C before use. For the h-BN/MWCNTs and HNT/MWCNT systems, the fillers were mechanically premixed according to the designed ratios prior to compounding. For the P34HB/h-BN/MWCNT system, a solution-based pre-dispersion method was employed. P34HB pellets were dissolved in dichloromethane to obtain a 10 wt% solution, followed by magnetic stirring at room temperature for 30 min until complete dissolution. Subsequently, h-BN and MWCNTs were added to the solution under continuous magnetic stirring for 2 h to ensure uniform dispersion. The mixture was then transferred to a fume hood and allowed to stand at room temperature for 24 h to evaporate the solvent. The resulting P34HB/h-BN/MWCNT composite was ground into a fine powder using a mortar and pestle and sieved through a 100-mesh screen. The prepared composite filler powder was sealed and stored for further use.

### 2.3. Sample Preparation

The basic rubber formulation is shown in Table 1. The filler formulations used are abbreviated as follows: h-BN as B_x_, MWCNTs as C_x_, P34HB as P_x_, and HNTs as H_x_, where x represents the parts per hundred rubber of each component.

**Figure 1:**
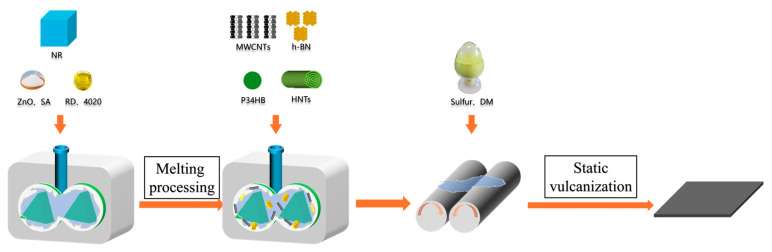
Flowchart illustrating the fabrication process of thermally conductive natural rubber composites.

The preparation process of the composite material is shown in Figure 1.

The internal mixer working temperature was set to 60 °C, and the rotor speed was set to 60 rpm. After reaching the set temperature, NR was added to the mixing chamber and masticated for 1 min. Subsequently, ZnO, SA, and antioxidants (RD and 4020) were added and mixed for 1.5 min. Thereafter, the corresponding fillers were added depending on the formulation, and the mixture was mixed for 5 min before being discharged.

After the compound cooled to below 50 °C, it was transferred to an open mill for refining. After the compound wrapped smoothly around the roller with no bubbles, sulfur and accelerator were added, triangular bags were folded six times, and the sheet was removed. The compound was allowed to stand for 24 h before vulcanization, which was conducted in a plate vulcanizing press under the following conditions: temperature: 150 °C, pressure: 13 MPa, and time: t_90_ + 2 min.

### 2.4. Characterization and Measurements

A field emission scanning electron microscope (S4800, Hitachi High-Technologies Corporation, Tokyo, Japan) was used to analyze the morphology of various functional fillers and composites. Before SEM observation, samples were sputter-coated with a 5 nm Au/Pd layer to enhance electrical conductivity.

For curing property testing, approximately 6 g of compound was weighed, sandwiched between glassine paper, and placed in the MDR (GT-M2000AN, Gotech Testing Machines Inc., Taichung, Taiwan). The test temperature was set to 150 °C, vibration frequency to 1.66 Hz, torque range from 0.01 to 22.6 N·m, and pressure to 0.36 MPa.

Thermal conductivity was measured using a thermal conductivity instrument (DTC 300 TA, NETZSCH Scientific Instruments Trading (Shanghai) Co., Ltd., Shanghai, China) at room temperature. Sample diameter was 50 mm, and thickness was 2 mm.

The dielectric properties were tested over a frequency range from 1 Hz to 1 MHz (10^0^–10^6^ Hz) at room temperature using a dielectric impedance spectrometer (Alpha-A, Novocontrol, Montabaur, Germany). The composite films were directly sandwiched between the two electrodes of the instrument without metalization. This non-metalized configuration avoids potential artifacts associated with sputtered electrodes, such as contact non-uniformity or induced interfacial states, ensuring that the measured dielectric response reflects the intrinsic properties of the composites.

Tensile properties were tested according to the national standard [32] using an AI-3000 tensile testing machine manufactured by Taiwan Gaotie Corporation (Taichung, Taiwan). The test specimen width was 4 mm, and the tensile speed was 300 mm/min. Five specimens were tested, and the average value was calculated.

Data analysis and plotting were performed using OriginPro 2021 (OriginLab Corporation, Northampton, MA, USA).

## 3. Results and Discussion

### 3.1. Vulcanization Properties

**Figure 2:**
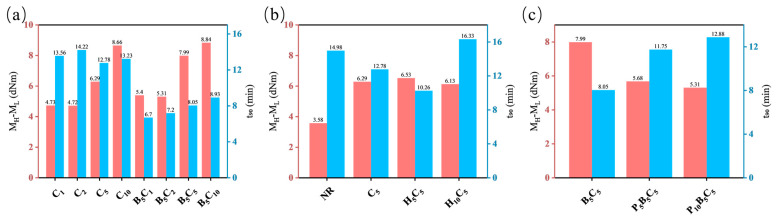
Curing characteristics (M_H_-M_L_ and t_90_) of different filler systems: (**a**) h-BN/MWCNTs, (**b**) HNTs/MWCNTs, and (**c**) P34HB/h-BN/MWCNTs.

The filler formulations and their curing characteristics parameters are listed in Table 2 and Figure 2, including minimum torque (M_L_), maximum torque (M_H_), torque difference (M_H_-M_L_), and optimum cure time (t_90_).

As shown in Figure 2a, compared with the pure CNT system, the h-BN/MWCNT hybrid system exhibits lower M_L_ values at all MWCNT loadings. This is attributed to the lubricating and physical barrier effects of h-BN, which disentangle the MWCNT agglomerates and reduce the initial viscosity. Meanwhile, the M_H_-M_L_ values of the hybrid system are generally higher than those of the pure CNT system at equivalent loadings, indicating increased crosslinking density. This enhancement comes from the improved dispersion of MWCNTs by h-BN, which exposes more active sites and promotes crosslinking with the rubber chains. Regarding the curing rate, the t_90_ values of the hybrid system are much lower, demonstrating an accelerating effect. This is due to the catalytic effect of uniformly dispersed MWCNTs on the vulcanization reaction, combined with the elimination of curing retardation caused by carbon nanotube agglomeration through the barrier and dispersion effects of h-BN. Thus, the two fillers synergistically achieve low viscosity, high crosslinking, and fast curing.

For the HNT/MWCNT tube–tube hybrid system, the curing behavior shows a non-monotonic dependence on HNT content. At an HNT loading of 5 phr, the M_H_-M_L_ value is slightly higher than that of C_5_, and the t_90_ is shortened from 12.78 min to 10.26 min. This is attributed to the weak interfacial interactions between the siloxane groups on the HNT surface and the MWCNTs, which optimize CNT dispersion, expose active sites, and improve vulcanization nucleation efficiency. When the HNT content is increased to 10 phr, the M_H_-M_L_ drops slightly below that of C_5_, while the t_90_ sharply increases to 16.33 min, even exceeding that of pure NR. This is mainly due to HNT agglomeration, which shields MWCNT active sites and forms a physical barrier that hinders curing agent diffusion, slowing crosslinking.

Introducing P34HB into the h-BN/MWCNT system significantly alters the curing characteristics (Figure 2c). Compared to B_5_C_5_, the addition of 5 phr P34HB reduces the M_H_-M_L_ value from 7.99 dNm to 5.68 dNm, and further to 5.31 dNm at 10 phr, indicating a continuous decline in crosslinking density. Concurrently, the t_90_ extends from 8.05 min to 11.75–12.88 min. This is ascribed to the phase separation between P34HB and NR, which disrupts the filler network and weakens the filler–matrix interactions. Moreover, the phase-separated interface hinders the uniform diffusion of curing agents, and the ester groups of P34HB may adsorb or undergo side reactions with active components of the curing system, collectively delaying vulcanization.

### 3.2. Microstructure Morphology

To investigate the dispersion state of the fillers, the cryogenically fractured surfaces of the samples were observed by SEM.

**Figure 3:**
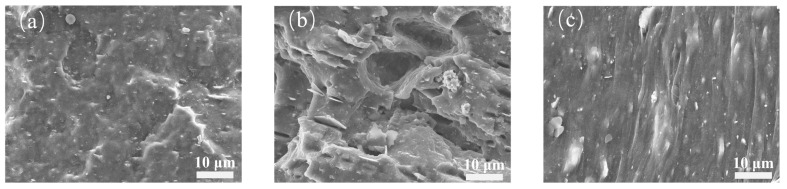
Fracture surface micrographs of h-BN/MWCNT hybrid composites (**a**) B_5_C_2_; (**b**) B_5_C_5_; and (**c**) B_5_C_10_.

As shown in Figure 3, at low MWCNT loading, the fracture surface of the B_5_C_2_ appears relatively smooth, with only scattered white spots and small protrusions on the rubber matrix and no large filler agglomerates. At this loading, the total filler content is low, resulting in limited reinforcement of the matrix. The fracture surface exhibits typical brittle fracture characteristics, with smooth crack propagation paths and weak interfacial interactions between the filler and the matrix.

As the MWCNT loading increases to 5 phr, B_5_C_5_ shows large-scale local agglomerates of MWCNTs and h-BN. During the fracture process, these agglomerates act as stress concentrators, inducing crack initiation and propagation, leaving distinct tearing ridges and voids on the fracture surface. Although the h-BN platelets still exert a certain physical barrier effect, the increased CNT content significantly enhances tube–tube interconnection and agglomeration. A physical crosslinking network begins to form within the matrix, and the fracture surface exhibits increased roughness and tearing characteristics, showing typical ductile fracture behavior, indicating that the fillers start to reinforce and toughen the matrix.

When the MWCNT loading is further increased to 10 phr, B_5_C_10_ displays a pronounced oriented fiber-like morphology on the fracture surface, with significant anisotropic stripes. Bright white agglomerates formed by the h-BN/MWCNT composite are uniformly distributed between the stripes. This fiber-like texture originates from the oriented flow marks left by large filler agglomerates dragged across the rubber surface under shear forces during mixing. This indicates that as the MWCNT content further increases, local agglomeration becomes more severe than B_5_C_5_, and matrix continuity is further reduced, with minor gaps and interfacial debonding between the stripes.

**Figure 4:**
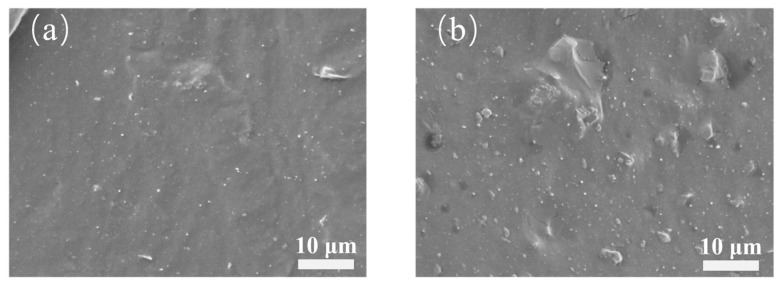
Fracture surface micrographs of HNT/MWCNT hybrid composites (**a**) H_5_C_5_; (**b**) H_10_C_5_.

As shown in Figure 4, comparison of the fracture surface morphologies of H_5_C_5_ and H_10_C_5_ reveals that at 5 phr HNTs, the fracture surface is smooth and continuous, with uniformly distributed fillers and no obvious agglomeration. The one-dimensional tubular HNTs and MWCNTs extend along the stress direction of the fracture, with HNTs intercalating between MWCNTs, effectively suppressing the self-entanglement of carbon nanotubes and achieving uniform filler dispersion within the matrix. The filler–rubber interface is tightly bonded, with no apparent debonding or gaps, indicating stable interfacial adhesion. As shown in Figure 4b, when the HNT content increases to 10 phr, the fracture surface becomes rougher, and local agglomeration of HNTs and MWCNTs occurs, forming clustered structures with uneven filler distribution. Within the agglomerates, the fillers are densely packed, while microscopic gaps exist at the interface with the rubber matrix, indicating weakened interfacial bonding. These observations demonstrate that as the HNT content increases from 5 phr to 10 phr, the microstructure of the tube–tube hybrid system undergoes significant changes, with the uniform dispersion state disrupted and interfacial bonding deteriorated.

**Figure 5:**
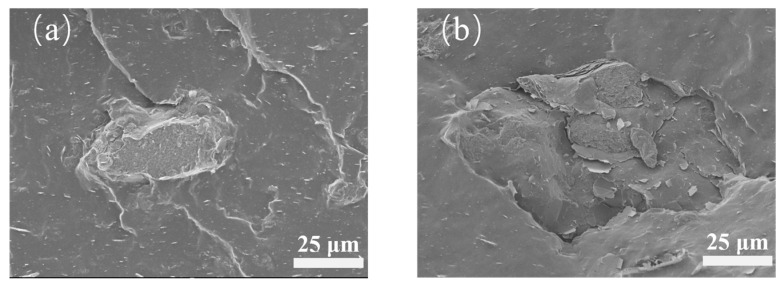
Fracture surface micrographs of P34HB/h-BN/MWCNT hybrid composites (**a**) P_5_B_5_C_5_; (**b**) P_10_B_5_C_5_.

As shown in Figure 5, in the fracture surface of P_5_B_5_C_5_, P34HB is embedded in the NR matrix as a discrete phase, exhibiting a typical macroscopic phase-separated morphology. Due to the thermodynamic incompatibility between NR and P34HB, P34HB undergoes rapid phase separation and coalesces during melt compounding, while h-BN and MWCNTs tend to encapsulate the P34HB phase, disrupting the filler network and compromising its continuity. At this P34HB loading, the degree of phase separation is relatively limited, and the interface between the P34HB phase and the NR matrix remains bonded, with the fracture surface dominated by brittle fracture characteristics.

When the P34HB content is increased to 10 phr, macroscopic phase separation becomes more pronounced. The P34HB domains grow in size, and the h-BN platelets and MWCNTs become more densely packed around the P34HB phase, forming distinct filler- and polymer-rich regions. The growth of the P34HB phase further disrupts the continuity of the NR matrix and intensifies the localized segregation of h-BN and MWCNTs, fragmenting the originally uniform thermal network. At higher magnification, microscopic gaps and debonding traces appear at the P34HB–NR interface, indicating weakened interfacial adhesion. The dense accumulation of fillers around the P34HB phase also weakens their interaction with the NR matrix, and the fracture surface remains predominantly brittle in nature.

### 3.3. Thermal Conductivity

**Figure 6:**
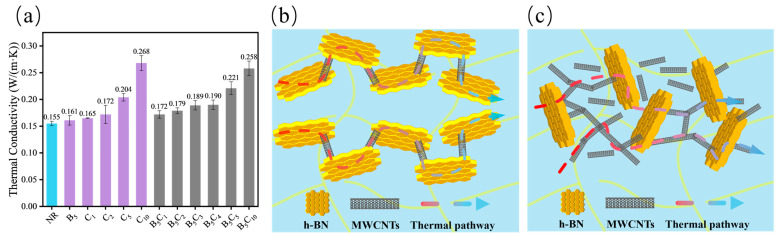
(**a**) Thermal conductivity of the h-BN/MWCNT hybrid system; (**b**,**c**) schematic illustration of thermal pathway evolution in the h-BN/MWCNT hybrid system at (**b**) low to medium and (**c**) high MWCNT loadings.

**Figure 7:**
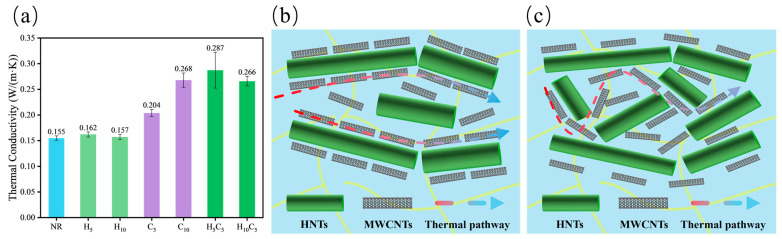
(**a**) Thermal conductivity of the HNT/MWCNT hybrid system; (**b**,**c**) schematic illustration of thermal pathways for (**b**) H_5_C_5_ and (**c**) H_10_C_5_.

**Figure 8:**
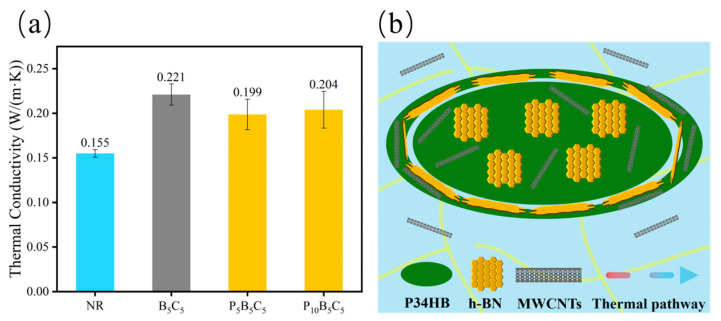
(**a**) Thermal conductivity of the P34HB/h-BN/MWCNT hybrid system; (**b**) schematic illustration of filler distribution.

The thermal conductivity of the composites was measured at room temperature to assess the efficiency of the constructed thermal networks. Figure 6, Figure 7 and Figure 8 present the thermal conductivity results and corresponding schematic illustrations of the thermal pathway evolution.

As shown in Figure 6a, for the h-BN/MWCNT hybrid system, the thermal conductivity of neat NR is 0.155 W/(m·K) and that of BN-5 (0.161 W/(m·K)) is close to neat NR, which is typical for low BN loadings in polymer composites [33]. When the MWCNT content increased from 1 phr to 5 phr, the thermal conductivity of the composite rose from 0.172 W/(m·K) to 0.221 W/(m·K), representing an 8.3% enhancement compared to CNT-5 at the same loading. The thermal conductivity of B_5_C_5_ is higher than both BN-5 and CNT-5, indicating a synergistic effect between h-BN and MWCNTs. This is attributed to the physical barrier and bridging effects of two-dimensional h-BN, which suppressed MWCNT agglomeration, filled the gaps in the carbon nanotube network, and facilitated the construction of a continuous three-dimensional thermal pathway (Figure 6b), achieving synergistic enhancement at low to medium loadings. When the MWCNT content was increased to 10 phr, the thermal conductivity reached 0.258 W/(m·K), which was comparable to that of CNT-10, indicating a weakened synergistic effect. This reflects that, at high loadings, the MWCNT network approaches saturation, the bridging contribution of h-BN becomes diluted, and h-BN may partially cut off direct contacts between carbon nanotubes (Figure 6c), thereby interfering with the original thermal pathways.

In contrast, the HNT/MWCNT tube–tube hybrid system exhibits superior synergistic enhancement in thermal conductivity. As shown in Figure 7a, the thermal conductivity of HNT-5 is 0.162 W/(m·K), and that of HNT-10 is 0.157 W/(m·K). Both values are only slightly above neat NR (0.155 W/(m·K)). This is mainly because HNTs have intrinsically low thermal conductivity (1–2 W/(m·K)) and large interfacial thermal resistance with the NR matrix [34]; the lower value at 10 phr HNTs likely arises from agglomeration and increased interfacial defects (Figure 4b). Despite the weak contribution of HNTs alone, H_5_C_5_ achieves a thermal conductivity of 0.287 W/(m·K), which is 40.6% higher than that of CNT-5 and even exceeds that of CNT-10. This improvement is attributed to the non-covalent interfacial interactions (hydrogen bonding and physical adsorption) between the siloxane groups on the HNT surface and the MWCNTs. Such moderate interactions induce the alignment of MWCNTs along the HNT long axis, creating low-resistance one-dimensional phonon pathways, while avoiding the local structural distortion and additional phonon scattering that overly strong covalent bonds may introduce. Additionally, the larger diameter and higher rigidity of HNTs allow them to compress MWCNTs into the interstitial spaces and induce oriented alignment, as illustrated in Figure 7b. This structure effectively disrupts the self-entanglement of carbon nanotubes and constructs oriented, low-resistance one-dimensional phonon transport pathways, contributing to enhanced thermal conductivity. When the HNT content is increased to 10 phr, the thermal conductivity decreases to 0.266 W/(m·K), comparable to that of CNT-10. This decline is primarily attributed to local agglomeration caused by the increased HNT content, which disrupts network continuity, as shown in Figure 7c. The agglomeration-induced protrusions and gaps observed in the fracture surfaces from the previous SEM section provide clear evidence of this structural deterioration.

As shown in Figure 8a, the introduction of P34HB into the h-BN/MWCNT system leads to a decrease in thermal conductivity. This is attributed to the macroscopic phase separation between P34HB and NR. As illustrated in Figure 8b, P34HB forms large agglomerates embedded in the matrix, while h-BN and MWCNTs tend to accumulate and encapsulate around the P34HB phase, disrupting the original filler network. On one hand, the intrinsically low thermal conductivity of P34HB dilutes the content of the highly thermally conductive components; on the other hand, the localized accumulation of fillers cuts off the phonon transport pathways, fragmenting the continuous thermal network. When the P34HB content increases from 5 phr to 10 phr, the thermal conductivity shows little further change, indicating that the disruption of the thermal network by phase separation approaches saturation. Nevertheless, the thermal conductivity of the hybrid system remains significantly higher than that of pure NR, suggesting that a portion of the fillers is not completely isolated during phase separation, and local phonon transport pathways are preserved, allowing the material to achieve good electrical insulation at the cost of some thermal performance.

### 3.4. Dielectric Properties

**Figure 9:**
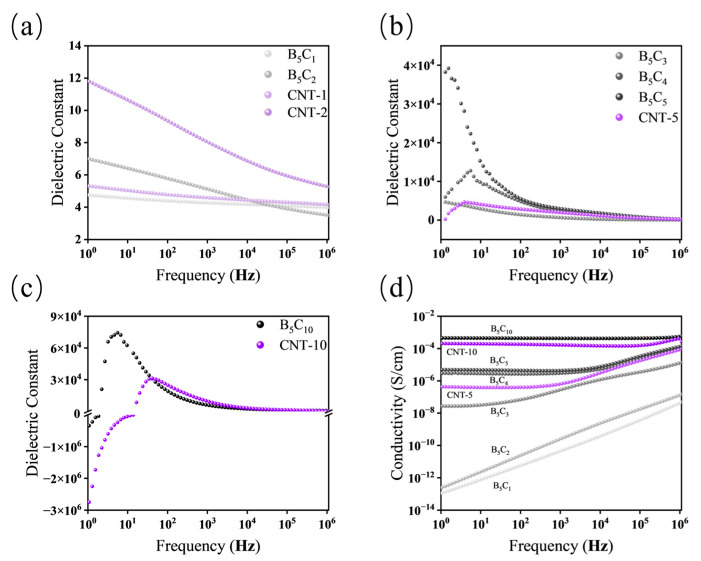
Frequency-dependent dielectric constant and conductivity of the h-BN/MWCNT hybrid system and pure CNT counterparts: (**a**) dielectric constant for B_5_C_1_, B_5_C_2_, CNT-1, and CNT-2; (**b**) dielectric constant for B_5_C_3_, B_5_C_4_, B_5_C_5_, and CNT-5; (**c**) dielectric constant for B_5_C_10_ and CNT-10; (**d**) conductivity for all formulations.

**Figure 10:**
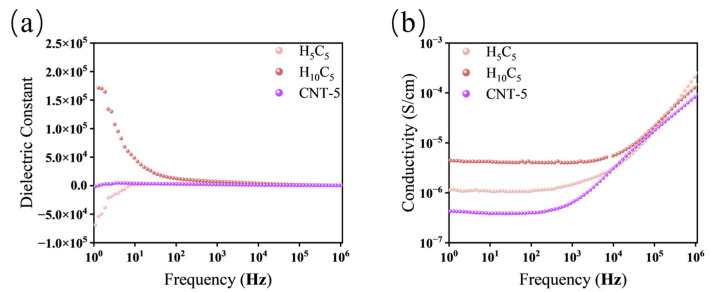
Frequency-dependent dielectric constant and conductivity of the HNT/MWCNT hybrid system: (**a**) dielectric constant; (**b**) conductivity.

**Figure 11:**
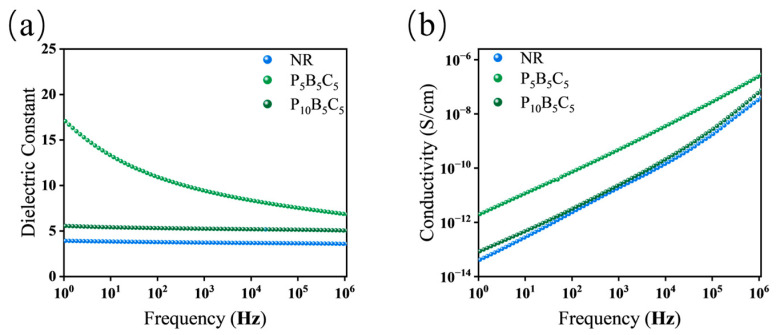
Frequency-dependent dielectric constant and conductivity of the P34HB/h-BN/MWCNT hybrid system: (**a**) dielectric constant; (**b**) conductivity.

The dielectric properties of the composites were investigated over a wide frequency range to understand the evolution of polarization and conduction mechanisms. Figure 9, Figure 10 and Figure 11 present the frequency-dependent dielectric constant and conductivity for different filler systems.

In the h-BN/MWCNT hybrid system, as the MWCNT content increases, the dielectric response and conductive behavior exhibit a transition from an insulating state to a conductive state, with the network topology significantly regulated by h-BN, as shown in Figure 9.

At low MWCNT loadings (1–3 phr), h-BN primarily acts as a physical barrier. The low-frequency dielectric constant and conductivity of B_5_C_1_ and B_5_C_2_ are lower than those of the corresponding pure CNT systems, indicating that the physical barrier effect of h-BN suppresses the strong interfacial polarization caused by MWCNT agglomeration and disrupts some conductive pathways. When the MWCNT content increases to 3 phr, B_5_C_3_ exhibits a conductivity of 5.0 × 10^−8^ S/cm, remaining in the insulating regime, with its dielectric constant increasing monotonically as frequency decreases, suggesting that MWCNTs are still isolated.

When the MWCNT content increased to 4 phr, the conductivity of B_5_C_4_ jumps from 5.0 × 10^−8^ S/cm to 5.0 × 10^−6^ S/cm, indicating that MWCNTs have started to form conductive pathways. However, its dielectric constant exhibits a peak at around 6–7 Hz followed by a decrease with decreasing frequency. This “rise-then-fall” behavior reflects competition between MWS polarization and leakage conduction: above a critical frequency, confined carriers cause MWS polarization and rising ε′; below it, carrier migration along loosely connected MWCNT pathways generates leakage current that consumes the polarization charges, reducing ε′.

When the MWCNT content increases to 5 phr, h-BN acts as a rigid insulating scaffold, constructing stable local networks. The conductivity of B_5_C_5_ increases to 7.0 × 10^−6^ S/cm, and its dielectric constant exhibits a slight inflection around 1 Hz, reaching 3.8 × 10^4^ at 1 Hz. In contrast, CNT-5 exhibits negative dielectric behavior in the low-frequency region. This phenomenon occurs when the conductive filler content exceeds the percolation threshold. The interconnected conductive network induces an equivalent inductance effect at low frequencies, leading to a negative real part of permittivity. This behavior is understood within the framework of MWS interfacial polarization combined with leakage conduction, rather than as a plasma oscillation of free charge carriers [28]. In B_5_C_5_, h-BN partitions MWCNTs into local dense networks, confining charge carriers and producing high conductivity and high dielectric constant without negative permittivity.

At high MWCNT loading, h-BN can no longer fully segregate the network, but still influences network topology through dispersion and path regulation. When the MWCNT content is increased to 10 phr, both B_5_C_10_ and CNT-10 exhibit negative dielectric behavior. However, compared to CNT-10, B_5_C_10_ shows a much higher peak dielectric constant, a lower transition frequency ω_p_ (from positive to negative permittivity), and higher conductivity across the entire frequency range. This indicates that the MWCNT content far exceeds the critical value, and h-BN can no longer fully segregate the network; instead, numerous MWCNTs interconnect within the gaps between h-BN platelets, forming a global conductive pathway. Nevertheless, h-BN still enhances local network density and interfacial polarization through its dispersing effect, while its insulating barrier shortens the effective migration paths of charge carriers. This reduced ω_p_ in B_5_C_10_ reflects the shortened effective migration paths of charge carriers resulting from the insulating barrier of h-BN. Meanwhile, the negative permittivity of CNT-10 was further examined as a function of oscillation voltage. As shown in Supplementary Figure S1, when the voltage increased from 1 V to 3 V, ω_p_ shifted from 19.9 Hz to 123.2 Hz. This shift is characteristic of a percolating conductive network, where higher voltages enhance leakage conduction and may induce local Joule heating, thereby modulating the equivalent inductance.

To further elucidate the charge transport mechanism, Cole–Cole plots and Jonscher power law fits were performed on three representative samples (B_5_C_2_, B_5_C_4_, and B_5_C_10_); the results are presented in Supplementary Figures S2 and S3 and Table S1. B_5_C_2_ exhibits a semicircular arc in the Cole–Cole plot, characteristic of MWS polarization below the percolation threshold. With increasing MWCNT content, B_5_C_4_ shows a distorted arc with a backward bend, indicating competition between polarization and leakage conduction near the percolation threshold. B_5_C_10_ displays a curve entering the negative dielectric constant region, confirming a fully percolated conductive network. The Jonscher exponent n, obtained from linear fits of log σ vs. log ω, decreases progressively from 1.01 for B_5_C_2_ (full frequency range, R^2^ = 0.9997) to 0.689 for B_5_C_4_ (10^4^–10^6^ Hz, R^2^ = 0.997) and further to 0.094 for B_5_C_10_ (10^5^–10^6^ Hz, R^2^ = 0.887). This monotonic decrease of n quantitatively demonstrates the transition from MWS polarization to hopping-dominated conduction with increasing MWCNT content.

As shown in Figure 10, H_5_C_5_ exhibits a positive dielectric constant at high frequencies, transitioning to negative around 6 Hz, with ω_p_ higher than that of CNT-5. The negative permittivity reaches −7 × 10^4^ at 1 Hz, far exceeding that of CNT-5, while the peak positive permittivity before the transition is lower. This arises from the regulation of MWCNT dispersion by HNTs through chemical adsorption and physical extrusion. This physical extrusion plays a dual role: in thermal conduction, it induces oriented alignment of carbon nanotubes to construct phonon transport pathways; in dielectric responses, it moderately disrupts the long-range conductive network, confining charge carriers to local regions. Meanwhile, the uniformly dispersed fillers form a more continuous conductive network, enhancing the equivalent inductance effect of the percolating network, thereby strengthening the negative permittivity response and shifting ω_p_ to higher values.

When the HNT content increases to 10 phr, H_10_C_5_ maintains a positive permittivity across the entire frequency range, with a higher low-frequency dielectric constant than CNT-5. This is attributed to HNT agglomeration disrupting the MWCNT conductive network while forming numerous interfaces that generate strong interfacial polarization. In terms of conductivity, H_10_C_5_ shows the highest value, originating from displacement current due to interfacial polarization, not charge carrier migration. Thermogravimetric analysis (TGA) of H10C5 reveals a mass loss of less than 0.2 wt% below 100 °C (Figure S4), confirming the absence of significant free water that could contribute to ionic conduction. Therefore, the high conductivity is attributed to the displacement current associated with its high dielectric constant, rather than to moisture or impurities. H_5_C_5_ exhibits intermediate conductivity due to its partially connected conductive network, while CNT-5 shows the lowest conductivity due to high contact resistance of entangled MWCNTs. These findings reveal that HNTs moderately disperse MWCNTs at low loadings but cause agglomeration at high loadings.

As can be seen from Figure 11, the introduction of P34HB exerts a fundamental impact on the dielectric and conductive properties of the h-BN/MWCNT system. The B_5_C_5_ sample exhibits a dielectric constant as high as 3.8 × 10^4^ at 1 Hz, demonstrating strong interfacial polarization. After the introduction of P34HB, the dielectric constant decreases to 17 for P_5_B_5_C_5_ and further drops to 5.5 for P_10_B_5_C_5_, approaching that of pure NR at 3.9. In terms of conductivity, B_5_C_5_ shows a value of 4.9 × 10^−6^ S/cm at 1 Hz, which decreases to 2.0 × 10^−12^ S/cm for P_5_B_5_C_5_ and further to 8.6 × 10^−14^ S/cm for P_10_B_5_C_5_, comparable to that of pure NR at 4.2 × 10^−14^ S/cm. These changes indicate that the high polarization and high conductivity characteristics of B_5_C_5_ are disrupted upon the introduction of P34HB, with the dielectric and conductive properties returning to near-insulating levels comparable to pure NR, and the disruption becomes more pronounced with increasing P34HB content.

This phenomenon arises from the phase separation between P34HB and NR during melt compounding, resulting in a dispersed island phase structure. h-BN and MWCNTs tend to distribute around the P34HB phase interfaces or become encapsulated within the P34HB phase, disrupting the originally platelet–tube synergistic network, significantly weakening the interfacial polarization effect, and cutting off the long-range migration paths of charge carriers. This contrast highlights the different requirements for electron and phonon transport: while electronic conduction requires a fully percolated network and is thus completely suppressed by phase separation, phonon transport can still occur through local filler-rich domains via local phonon conduction through closely spaced but non-percolating filler networks, explaining why the thermal conductivity remains higher than that of pure NR despite the disrupted filler network. This result corroborates the observed reduction in thermal conductivity, confirming the destructive effect of phase separation.

### 3.5. Mechanical Properties

**Figure 12:**
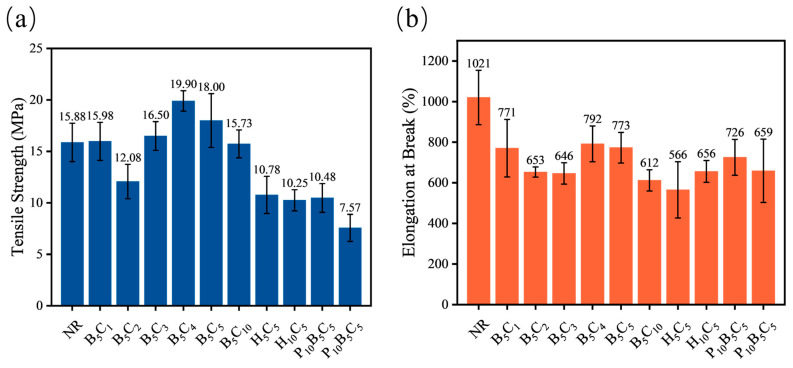
Mechanical properties of h-BN/MWCNT, HNTs/MWCNT, and P34HB/h-BN/MWCNT composites: (**a**) tensile strength, (**b**) elongation at break.

The mechanical performance of the composites was evaluated through tensile testing to correlate the filler network structure with reinforcement efficiency. Figure 12 shows the tensile strength and elongation at break for all formulations.

As shown in Figure 12, the tensile strength of the h-BN/MWCNT system shows a non-monotonic trend with increasing MWCNT content, first decreasing, then increasing, and finally decreasing. At low loadings, B_5_C_1_ is comparable to pure NR, while B_5_C_2_ decreases significantly due to local weak interface regions between MWCNT and h-BN that partially disrupt the rubber network. At 3–5 phr, tensile strength recovers, with B_5_C_4_ reaching the peak and B_5_C_5_ remaining high, as sufficient MWCNTs connect h-BN platelets to form a synergistic reinforcing network that enhances stress transfer. At 10 phr, tensile strength declines due to excessive agglomeration and network disruption. Elongation at break generally decreases with filler addition, but B_5_C_4_ and B_5_C_5_ maintain relatively high values while achieving enhanced strength, indicating that a stable filler network improves stress distribution.

In the HNT/MWCNT system, the tensile strength and elongation at break of H_5_C_5_ and H_10_C_5_ are significantly lower than those of the h-BN/MWCNT system and even lower than those of pure NR. Although HNTs exhibit some physical compatibility with NR, there is a lack of strong chemical bonding and mechanical interlocking between them, resulting in intrinsically weak interfacial adhesion. This unique interfacial state—good dispersion but weak bonding—determines that the HNT/MWCNT system can only form a weakly connected physical contact network. It is worth noting that the “physical extrusion” effect of HNTs on MWCNTs, while beneficial for dispersing carbon nanotubes and inducing their alignment, may also promote the formation of micro-defects or voids within the NR matrix at higher HNT loadings. As shown in Figure 4b, local agglomerates of HNTs and MWCNTs appear in the H_10_C_5_ sample, with microscopic gaps and debonding traces visible at the filler–rubber interface. These micro-defects act as stress concentrators and crack initiation sites during tensile deformation. While such a network enables relatively efficient phonon transport through close physical proximity effects, it fails to meet the strong interfacial coupling requirements for load bearing, and the incorporation of MWCNTs further disrupts the strain-induced crystallization of NR, leading to mechanical properties inferior to those of pure NR.

After the introduction of P34HB, the mechanical properties of the h-BN/MWCNT system deteriorate sharply. The tensile strength of P_5_B_5_C_5_ decreases significantly compared to B_5_C_5_, and further drops to 7.58 MPa for P_10_B_5_C_5_, accompanied by a corresponding reduction in elongation at break. This is attributed to the macroscopic phase separation caused by the polarity difference and thermodynamic incompatibility between P34HB and NR, which leads to the formation of micro-defects. Meanwhile, the dispersed P34HB phase dilutes the effective crosslinking density of the rubber matrix and interferes with the construction and functionality of the h-BN/MWCNT reinforcing network, thereby hindering stress transfer within the material.

## 4. Discussion

### 4.1. Unified Mechanistic Model

The results in Section 3.1, Section 3.2, Section 3.3 and Section 3.4 reveal a relationship between filler geometry, network topology, and overall performance. Two-dimensional h-BN forms an isolated structure; one-dimensional HNTs and MWCNTs interpenetrate and align via hydrogen bonding to form a percolating network; immiscible P34HB and NR undergo phase separation, forcing fillers into an island-like distribution. These topologies, together with interfacial bonding strength, govern thermal, dielectric, and mechanical performance.

In the isolated network (platelet–tube), h-BN partitions MWCNTs into local regions, creating a “locally dense, globally constrained” topology. Charge carriers are confined, giving a high dielectric constant (3.8 × 10^4^ at 1 Hz), no negative permittivity, and moderate conductivity (7.0 × 10^−6^ S/cm). Thermal conductivity reaches 0.221 W/(m·K), and the mechanical properties are close to those of pure NR. In the percolating network (tube–tube), continuous phonon pathways yield the highest thermal conductivity (0.287 W/(m·K), 40.6% above CNT-5). However, the interfacial bonding is limited to hydrogen bonding and physical adsorption, leading to poor stress transfer and a decrease in tensile strength of about 30% relative to pure NR. In the island-like network (phase-separated), filler segregation into the P34HB phase cuts off long-range conductive pathways, dropping conductivity to near insulation (∼10^−14^ S/cm) while preserving local thermal pathways (κ > pure NR). The phase interfaces become mechanically weak, causing a tensile strength loss of more than 50%.

Interfacial bonding strength is equally critical. Strong covalent or polar interfaces favor stress transfer and good mechanical properties but may hinder filler alignment. Weak non-covalent interfaces promote filler orientation and network construction at the expense of mechanical properties (as seen in the tube–tube system). Macroscopic phase interfaces enable a “conductivity-off, thermal-on” decoupled state but create mechanical weaknesses.

### 4.2. Comparison with Literature

To assess the positioning of the different filler strategies in this work, three representative BN/MWCNT hybrid systems from the literature are selected for comparison (Table 3). These systems differ in matrix, processing method, and network architecture, offering complementary perspectives to our work.

Compared with the literature (Table 3): Wu et al. [35] achieved 0.66 W/(m·K) and a dielectric constant of 123 using a segregated double network with continuous h-BN layers. Our B_5_C_5_ lacks such continuous layers, giving lower thermal conductivity of 0.221 W/(m·K) but a much higher dielectric constant of about 3.8 × 10^4^, which is advantageous for energy storage. Zhang et al. [36] chemically bonded h-BN to an epoxy vitrimer matrix via dynamic transesterification, reaching 0.83 W/(m·K) at low filler loading; our simple physical mixing avoids complex chemistry, offering better scalability, but leaves higher thermal resistance. Xue et al. [37] obtained 0.279 W/(m·K) by bridging BN platelets with CNTs in silicone rubber. Our H_5_C_5_ matches this value through HNT-induced MWCNT alignment, confirming the tube–tube strategy’s effectiveness, though HNT’s low intrinsic conductivity limits further improvement. Finally, our P_10_B_5_C_5_ achieves a unique “conductivity-off, thermal-on” state with electrical conductivity about 10^−14^ S/cm near insulation and thermal conductivity above that of pure NR via phase separation, a decoupled behavior not seen in the three reference works. The systematic comparison of three distinct dimensional strategies within the same matrix is the unique contribution of this work.

### 4.3. Limitations and Future Perspectives

Each filler strategy has inherent limitations. For the platelet–tube system, the enhancement in thermal conductivity is limited, and the insulating scaffold fails to fully isolate the conductive network at high MWCNT loadings. For the tube–tube system, the weak non-covalent interfacial bonding leads to mechanical properties inferior to pure NR, restricting its use in load-bearing applications. For the phase-separated system, the introduction of P34HB causes a severe drop in tensile strength, making compatibilization necessary for practical utility.

To address these limitations, future work should focus on interface engineering (e.g., silane functionalization) and morphology control (e.g., compatibilizers) to improve mechanical properties, while network optimization (e.g., 3D continuous networks or external field alignment) and long-term reliability evaluation are also needed.

## 5. Conclusions

This study demonstrates three multi-dimensional filler strategies in natural rubber composites. The h-BN/MWCNT platelet–tube system forms a locally conductive yet globally constrained network, achieving a high dielectric constant without negative permittivity, moderate thermal conductivity, and reasonably good mechanical properties. The HNT/MWCNT tube–tube system creates oriented one-dimensional phonon pathways via hydrogen bonding, giving the highest thermal conductivity at the cost of mechanical properties due to weak interfacial bonding. The P34HB/h-BN/MWCNT phase-separated system achieves a unique “conductivity-off, thermal-on” decoupled state, with conductivity dropping to near-insulating levels and thermal conductivity remaining above that of pure NR, yet tensile strength falls by more than half. These results reveal that filler geometry dictates network topology, which together with interfacial interactions determines the trade-offs among thermal, dielectric, and mechanical performance.

Nevertheless, future work should focus on further enhancing thermal conductivity through network optimization, filler functionalization, and external field alignment, while also evaluating long-term reliability under cyclic loading and thermal aging conditions.

## Figures and Tables

**Table 1:** The basic formula of the experiment.

Component	Concentration (phr)
Natural rubber (NR)	100
Zinc oxide (ZnO)	5
Stearic acid (SA)	3
Sulfur (S)	2.5
Dibenzothiazole disulfide (DM)	0.6
N-Isopropyl-N′-phenyl diphenylamine (4020)	1.6
2,2,4-Trimethyl-1,2-dihydroquinoline polymer (RD)	0.5

**Table 2:** Vulcanization parameters of all composite formulations.

Sample	M_L_ (dNm)	M_H_ (dNm)	M_H_-M_L_ (dNm)	t_90_ (min)
NR	0.56	4.14	3.58	14.98
B_5_	0.53	4.91	4.38	14.10
C_1_	0.48	5.21	4.73	13.56
C_2_	0.58	5.30	4.72	14.22
C_5_	0.86	7.15	6.29	12.78
C_10_	1.51	10.17	8.66	13.23
B_5_C_1_	0.17	5.57	5.40	6.70
B_5_C_2_	0.07	5.38	5.31	7.20
B_5_C_3_	0.37	7.00	6.63	6.97
B_5_C_4_	0.36	7.50	7.14	7.39
B_5_C_5_	0.48	8.47	7.99	8.05
B_5_C_10_	0.62	9.46	8.84	8.93
H_5_C_5_	0.80	7.33	6.53	10.26
H_10_C_5_	0.85	6.98	6.13	16.33
P_5_B_5_C_5_	0.51	6.19	5.68	11.75
P_10_B_5_C_5_	0.52	5.83	5.31	12.88

**Table 3:** Comparison with literature hybrid composites.

System	Loading	Thermal Conductivity (W/(m·K))	Dielectric Constant
PS/MWCNT@h-BN [35]	1 + 10 vol% (MWCNT + h-BN)	0.66	123 (100 Hz)
Epoxy vitrimer/MWCNT/h-BN [36]	1 + 8 wt% (MWCNT + h-BN)	0.83	—
Silicone rubber/CNT/BN [37]	0.25 vol% CNT + 30 phr BN	0.279	~2.9 (10 Hz)
B_5_C_5_	5 + 5 phr (h-BN + MWCNT)	0.221	~3.8 × 10^4^ (1 Hz)
H_5_C_5_	5 + 5 phr (HNT + MWCNT)	0.287	Positive → negative (6 Hz)
P_10_B_5_C_5_	10 + 5 + 5 phr (P34HB + h-BN + MWCNT)	0.204	~5.5 (1 Hz)

## Data Availability

The original contributions presented in this study are included in the article/Supplementary Material. Further inquiries can be directed to the corresponding author.

## Supplementary Materials

The following supporting information can be downloaded at: https://www.mdpi.com/article/10.3390/polym18091074/s1. Figure S1. Frequency-dependent real permittivity of CNT-10 under different oscillation voltages (1 V, 3 V); Figure S2. Cole-Cole plots (ε′′ vs ε′) of representative samples; Figure S3. Jonscher power-law fits (log σ vs log ω) for the same three samples; Figure S4. Thermogravimetric analysis (TGA) curve of the H10C5 composite. The mass loss below 100 °C is less than 0.2 wt%, indicating negligible free water content. Table S1. Jonscher power-law fitting parameters for representative samples.

## Abbreviations

The following abbreviations are used in this manuscript:

NR	Natural rubber
h-BN	Hexagonal boron nitride
HNTs	Halloysite nanotubes
P34HB	Poly(3-hydroxybutyrate-co-4-hydroxyvalerate)
MWCNTs	Multi-walled carbon nanotubes
SEM	Scanning electron microscope
MDR	Moving Die Rheometer
M_L_	Minimum torque
M_H_	Maximum torque
t_90_	Optimum cure time
ω_p_	Plasma frequency
σ	Conductivity
ω	Angular frequency
ε′	Real part of permittivity (dielectric constant)
ε″	Imaginary part of permittivity (dielectric loss)
n	Jonscher exponent
R^2^	Coefficient of determination
