# Supplementary material for: Multi-Dimensional Filler Design for Enhanced Thermal Conductivity and Tunable Dielectric Properties in Natural Rubber Composites
Source: Polymers (Basel). 2026 Apr 29;18(9):1074. doi: 10.3390/polym18091074 (PMC13165945; doi:10.3390/polym18091074)
Supplement: Supplementary file 1 [file polymers-18-01074-s001.zip › polymers-4258321-supplementary.pdf]

polymers

MDPI

Article

Multi-Dimensional Filler Design for Enhanced Thermal Conductivity and Tunable Dielectric Properties in Natural Rubber Composites

Yu Li [1], Qihan Cui [1], Yining Wang [1], Yuanqin Gao [1], Xianhua Hu [1], Xueqing Liu [2], Yumin Xia [3], Lan Cao [1] and Yuwei Chen [1,*]

[1] Key Laboratory of Advanced Rubber Material, Ministry of Education, Qingdao University of Science and Technology, Qingdao 266042, China; m17862186395@163.com (Y.L.); qihancui@163.com (Q.C.); 18905438518@163.com (Y.W.); 19853202317@163.com (Y.G.);13969526201@163.com (X.H.); lancao@qust.edu.cn (L.C.)

[2] Key Laboratory of Optoelectronic Chemical Materials and Devices, Ministry of Education and Flexible Display Materials and Technology Co-Innovation Centre of Hubei Province, Jianghan University, Wuhan 430056, China; xqliu@jhun.edu.cn

[3] State Key Laboratory for Modification of Chemical Fibers and Polymer Materials, College of Materials Science and Engineering, Donghua University, Shanghai 201600, China; xym@dhu.edu.cn

* Correspondence: yuweichen@qust.edu.cn

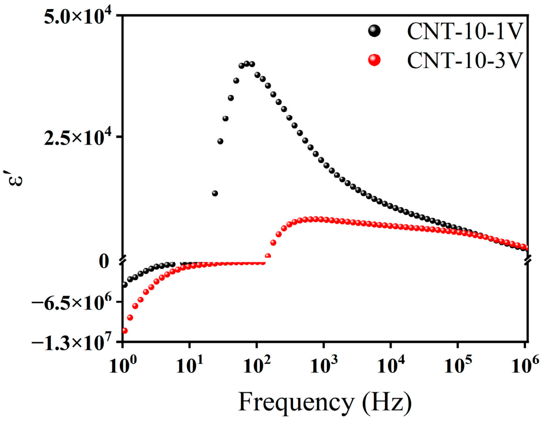

Figure S1. Frequency-dependent real permittivity of CNT-10 under different oscillation voltages (1 V, 3 V)

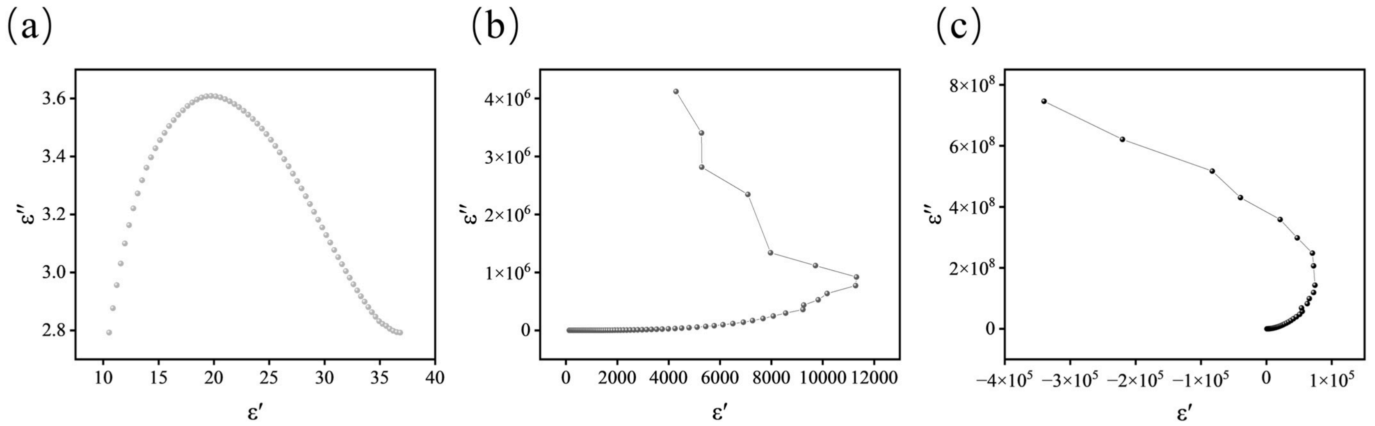

Figure S2. Cole-Cole plots (ε″ vs ε′) of representative samples: (a) B5C2, showing a semicircular arc characteristic of MWS polarization below the percolation threshold; (b) B5C4, showing a distorted arc with a backward bend, indicating competition between polarization and leakage conduction

 https://doi.org/10.3390/polym18091074

near the threshold; (c) B5C10, showing a curve entering the negative ε′ region, confirming a fully percolated conductive network.

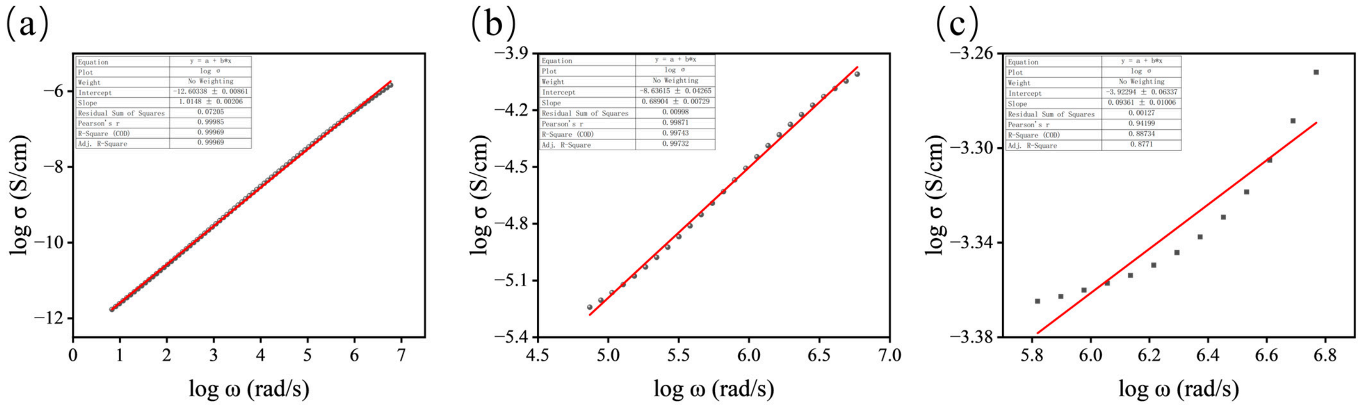

Figure S3. Jonscher power-law fits (log σ vs log ω) for the same three samples: (a) B5C2 over the full frequency range (1 Hz – 1 MHz); (b) B5C4 in the high-frequency region (10^4 Hz – 1 MHz); (c) B5C10 in the highest frequency region (10^5 Hz – 1 MHz). Solid lines represent linear fits, and the derived exponent n and R^2 are indicated in each panel. The progressive decrease of n from ~1 to ~0.09 quantitatively demonstrates the transition from MWS polarization to hopping-dominated conduction.

Table S1. Jonscher power-law fitting parameters for representative samples.

Sample	Fitting frequency range (Hz)	n	R^2
B_5C_2	$10^0 – 10^6$	1.014	0.9997
B_5C_4	$10^4 – 10^6$	0.689	0.997
B_5C_{10}	$10^5 – 10^6$	0.094	0.887

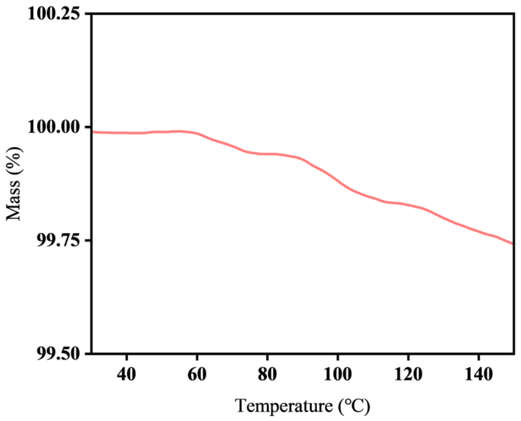

Figure S4. Thermogravimetric analysis (TGA) curve of the H10C5 composite. The mass loss below 100 °C is less than 0.2 wt%, indicating negligible free water content.

https://doi.org/10.3390/polym18091074
